# Death domain-associated protein (DAXX) expression is associated with poor survival in metastatic high-grade serous carcinoma

**DOI:** 10.1007/s00428-020-02842-4

**Published:** 2020-06-12

**Authors:** Ben Davidson, Erin McFadden, Arild Holth, Marta Brunetti, Vivi Ann Flørenes

**Affiliations:** 1 Department of Pathology, Norwegian Radium Hospital, Oslo University Hospital, Montebello, N-0310 Oslo, Norway; 2 Faculty of Medicine, Institute of Clinical Medicine, University of Oslo, N-0316 Oslo, Norway; 3 Section for Cancer Cytogenetics, Institute for Cancer Genetics and Informatics, The Norwegian Radium Hospital, Oslo University Hospital, N-0310 Oslo, Norway

**Keywords:** DAXX, ATRX, Immunohistochemistry, Western blotting, High-grade serous carcinoma, Effusion

## Abstract

The objective of this study was to analyze the expression and clinical role of mitosis regulators α-thalassemia/mental retardation syndrome X-linked (ATRX) and death-domain-associated protein (DAXX) in metastatic high-grade serous carcinoma (HGSC). ATRX and DAXX protein expression by immunohistochemistry was analyzed in 400 HGSC effusions. DAXX expression was additionally studied in 15 cancer cell lines, including 4 ovarian carcinoma lines, and in 81 of the 400 HGSC effusions using Western blotting. ATRX and DAXX were expressed in HGSC cells in 386/400 (96%) and 348/400 (87%) effusions, respectively. Western blotting showed DAXX expression in all 15 cell lines and in 70/81 (86%) HGSC effusions. DAXX expression by immunohistochemistry was higher in pleural compared to peritoneal effusions (*p* = 0.006) and in post-chemotherapy compared to pre-chemotherapy effusions (*p* = 0.004), and its expression was significantly associated with poor overall survival in univariate of the entire cohort (*p* = 0.014), as well as analysis limited to chemo-naïve effusions tapped at diagnosis (*p* = 0.038). The former association retained its prognostic role in Cox multivariate survival analysis (*p* = 0.011). ATRX expression was unrelated to clinicopathologic parameters or survival. In conclusion, DAXX is associated with disease progression and could be a prognostic marker in metastatic HGSC. Silencing this molecule may have therapeutic relevance in this cancer.

## Introduction

Ovarian cancer, consisting mainly of ovarian carcinoma (OC), is the 7th most common cancer and the 8th most common cause of cancer death in women. In 2018, 239,000 women were diagnosed with this disease and 152,000 deaths occurred globally [1]. Overall survival (OS) is currently longer than previously, with approximately 45% of patients alive at 5 years, due to improved surgery and chemotherapy protocols, as well as targeted therapy. However, this figure is true for all histological types combined. In high-grade serous carcinoma (HGSC), the most common and aggressive type of OC, diagnosis is often at advanced-stage (FIGO stage III–IV) and death-of-disease occurs in the majority of patients [1]. HGSC develops most frequently in the fallopian tube and metastasizes widely within the peritoneal cavity, including the formation of malignant ascites, as well as to the pleural space. OC cells in effusions cannot be surgically eradicated, are anoikis-resistant, and possess cancer stem cell characteristics that facilitate chemoresistance [2]. Better understanding of the molecular profile of cancer cells in effusions is therefore an important challenge.

Eukaryotic DNA is wrapped around histones and is post-translationally modified to regulate transcription. Repetitive elements in the genome, termed tandem repeats, are organized in a condensed form termed heterochromatin, present in retrotransposons, pericentric heterochromatin, and telomeres, in order to avoid aberrant transcription. DNA synthesis during S-phase requires deposition of newly synthesized H3.1 and H3.2 canonical histones, which are S-phase-specific. In contrast, H3.3 histone is replication-independent and present throughout the cell cycle. Deposition of the latter requires chaperones, which may be either the HIRA complex or the ATRX/DAXX complex [3, 4].

ATRX is a chromatin remodeling protein belonging to the SNF2 sub-group of the SWI/SNF family, encoded by a gene on the long arm of the X-chromosome (Xq21.1). It has a crucial role in the development of organs from all 3 germ cell layers, as evidenced by the fact that mutations in it are the sole cause for ATRX (α-thalassemia, mental retardation, X-linked) syndrome. *ATRX* mutations are also found in different cancers, including glioma, neuroblastoma, pancreatic neuroendocrine tumors, and childhood osteosarcoma [4, 5]. Additionally, reduced ATRX expression has been reported in melanomas and sarcomas, the latter including uterine leiomyosarcoma [4–7]. Loss of ATRX results in DNA damage, genomic instability, and telomeric dysfunction [4].

DAXX (death domain-associated protein) was initially identified as a Fas-binding protein inducing apoptosis via JNK (Jun N-terminal kinase), but this role was subsequently questioned and the cellular localization of this protein has been shown to be nuclear, in accord with its observed role as transcriptional regulator. As with ATRX, DAXX mutations have been found in glioma and pancreatic neuroendocrine tumors [4].

Loss of ATRX and DAXX is strongly related to alternative lengthening of telomeres (ALT), a process characteristic of cancer cells in which telomere length is retained through a non-telomerase-dependent mechanism [8].

DAXX has been reported to have a tumor-promoting effect in vitro and in vivo in experimental models of OC, with increase in proliferation, survival, colony formation, and migration [9, 10]. It was further shown to be hypomethylated in chemoresistant compared to sensitive OC xenografts [11]. Analysis of the expression and clinical relevance of DAXX in patient material is to date limited to a single study of primary OC of various histotypes in which *DAXX* mRNA levels were not significantly related to survival [12]. To the best of our knowledge, the clinical role of ATRX has not been investigated in this cancer to date.

The objective of the present study was to assess the expression and clinical relevance of ATRX and DAXX in a large cohort of patients with HGSC effusions, the majority diagnosed at FIGO stage III–IV.

## Material and methods

### Patients and specimens

HGSC effusions (*n* = 400; 343 peritoneal, 57 pleural) from 400 patients were submitted to the Department of Pathology at the Norwegian Radium Hospital during the period of 1998 to 2015. Effusions were centrifuged immediately after tapping, and cell pellets were used for preparation of cell blocks using the thrombin clot protocol. Cell pellets were additionally frozen at − 70 °C in equal amounts of RPMI 1640 medium (GIBCO-Invitrogen, Carlsbad, CA) containing 50% fetal calf serum (PAA Laboratories GmbH, Pasching, Austria) and 20% dimethylsulfoxide (Merck KGaA, Darmstadt, Germany). Tumor cell content in all effusions studied by Western blotting was > 50%, based on assessment of cytology smears and H&E sections from the above cell blocks. Clinicopathologic data are detailed in Table 1. Informed consent was obtained according to national and institutional guidelines. Study approval was given by the Regional Committee for Medical Research Ethics in Norway.

**Table 1:** Clinicopathologic parameters of the HGSC effusion cohort (400 patients)

Parameter		Distribution
Age (mean)		23–88 years (63)
FIGO stage		
I		3
II		6
III		235
IV		151
NA		5
Residual disease		
Primary debulking surgery (*n* = 210)	0 cm	31
	≤ 1 cm	86
	> 1 cm	93
Interval debulking surgery (*n* = 109)	0 cm	30
	≤ 1 cm	48
	> 1 cm	31
NA		81
CA 125 at diagnosis (range; median)		10–62,400 (1257)^a^
Chemoresponse after primary treatment		
CR		183
PR		99
SD		30
PD		39
NA^b^		49

*NA*, not available; *CR*, complete response; *PR*, partial response; *SD*, stable disease; *PD*, progressive disease

^a^Available for 314 patients

^b^Not available (missing data or disease response after chemotherapy could not be evaluated because of normalized CA 125 after primary surgery or missing CA 125 information and no residual tumor)

### Immunohistochemistry

Formalin-fixed, paraffin-embedded sections from 400 HGSC effusions were analyzed for ATRX and DAXX protein expression using the Dako EnVision Flex + System (K8012; Dako, Glostrup, Denmark). The ATRX antibody was a mouse monoclonal antibody purchased from Novus Biologicals (cat # NBP2–52938, clone CL0537; Littleton, CO). The DAXX antibody was a rabbit polyclonal antibody purchased from Sigma-Aldrich (cat # HPA008736; St. Louis, MO; Powered by Atlas Antibodies, Stockholm, Sweden). Both antibodies were applied at a 1:500 dilution following antigen retrieval in Dako HpH (pH 9.0) solution.

Following deparaffinization, sections were treated with EnVision™ Flex + mouse linker (15 min) and EnVision™ Flex/HRP enzyme (30 min) and stained for 10 min with 3′3-diaminobenzidine tetrahydrochloride (DAB), counterstained with hematoxylin, and dehydrated and mounted in Richard-Allan Scientific Cyto seal XYL (Thermo Fisher Scientific, Waltham, MA). Positive controls consisted of normal testis. In the ATRX negative control, the primary antibody was replaced with isotype-specific mouse myeloma protein diluted to the same concentration as the primary antibody. The DAXX negative controls were incubated with rabbit serum.

Immunohistochemistry (IHC) scoring: Nuclear staining was scored by an experienced cytopathologist (BD), using a 0–4 scale as follows: 0 = no staining, 1 = 1–5%, 2 = 6–25%, 3 = 26–75%, 4 = 76–100% of tumor cells.

### Western blotting

Protein lysates from 81 of the 400 HGSC effusions were analyzed for DAXX protein expression by Western blotting (WB). Effusions were thawed, washed in phosphate-buffered saline, and lysed in lysis buffer (1% NP-40, 10% glycerol, 20 mM Tris HCl, pH = 7.5, 137 mM NaCl, 100 mM NaF, 1 mM sodium vanadate, 1 mM PMSF, 0.02 mg/ml each of aprotinin, leupeptin, and pepstatin, and 10 μL/ml each of phosphatase inhibitor cocktails I and II, the latter purchased from Sigma-Aldrich). Lysates were sonicated, and after centrifugation, the supernatant was collected and protein content was evaluated by the Bradford assay (Bio-Rad Laboratories, Hercules, CA). A total of 25 μg from each sample was separated by sodium dodecyl sulfate-polyacrylamide gel electrophoresis (SDS-PAGE). Proteins were transferred to PVDF immobile membranes (Millipore, Bedford, MA). Membranes were blocked in 5% nonfat dry milk, freshly made in 20 mmol/L Tris HCl, pH 7.6; 0.136 mol/L NaCl; 0.05% polysorbate (Tween) (TBST), and subsequently hybridized with the same DAXX antibody used in the IHC analysis, at 1:1000 dilution in 5% Bovine Serum Albumin (Sigma Aldrich) in 0.1% TBST, overnight at 4 °C. Thereafter, the blots were washed 3 times for 10 min in TBST and incubated for 60 min at room temperature with rabbit horseradish peroxidase-conjugated secondary antibodies (1:5000; Promega, Madison, WI) diluted in 5% non-fat dry milk in TBST. Immunoreactivity was detected using the Supersignal West Dura (Thermo Fisher Scientific) and visualized using Syngene G:box chemi XRQ (Syngene, Cambridge, UK). To ensure even loading, filters were hybridized with ERK2 polyclonal rabbit antibody (Santa Cruz Biotechnology, Santa Cruz, CA) 1:1000 in 5% non-fat dry milk.

### Statistical analysis

Statistical analysis was performed applying the SPSS-PC package (Version 25). Probability of < 0.05 was considered statistically significant. The Mann-Whitney *U* test or the Kruskal-Wallis H test was applied to analysis of the association between ATRX and DAXX protein expression by IHC and clinicopathologic parameters (for 2-tier or 3-tier analyses, respectively). For this analysis, clinicopathologic parameters were grouped as follows: age, ≤ 60 vs. >60 years; effusion site, peritoneal vs. pleural; FIGO stage, III vs. IV; chemotherapy status, pre- vs. post-chemotherapy specimens; residual disease (RD) volume, 0 cm vs. ≤ 1 cm vs. > 1 cm; response to chemotherapy, complete response vs. partial response/stable disease/progressive disease. Progression-free survival (PFS) and OS were calculated from the date of the last chemotherapy treatment/diagnosis to the date of recurrence/death or last follow-up, respectively. Univariate survival analyses of PFS and OS were executed using the Kaplan-Meier method and log-rank test. Multivariate survival analysis was executed using the Cox Regression Model. Platinum resistance was defined as PFS ≤ 6 months according to guidelines published by the Gynecologic Oncology Group (GOG) and progressive disease or recurrence was evaluated by the *Response Evaluation Criteria In Solid Tumors* (RECIST) criteria.

## Results

### ATRX and DAXX are frequently expressed in HGSC effusions

**Fig. 1:**
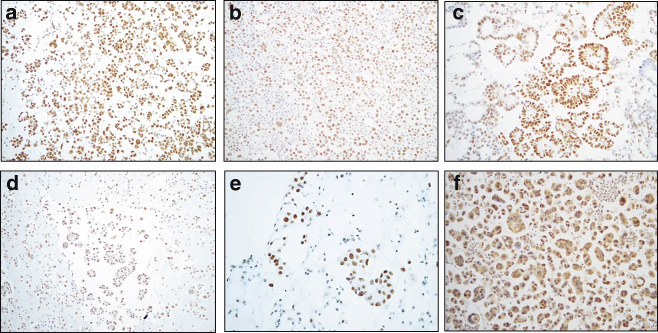
Immunohistochemistry: **a**–**c** Nuclear expression of ATRX in 3 effusion specimens; **d**–**f** nuclear expression of DAXX in 3 effusion specimens. Cytoplasmic staining is additionally seen in the tumor in Fig. 1f

ATRX and DAXX were expressed in HGSC cells in 386/400 (96%) and 348/400 (87%) effusions, respectively. Staining was predominantly nuclear, though few tumors showed additionally cytoplasmic DAXX expression (Fig. 1). Nuclear staining extent was as follows: ATRX: score = 0: 14; score = 1: 39; score = 2: 39; score = 3: 172; score = 4: 136 specimens; DAXX: score = 0: 52; score = 1: 120; score = 2: 48; score = 3: 142; score = 4: 38 specimens.

**Fig. 2:**
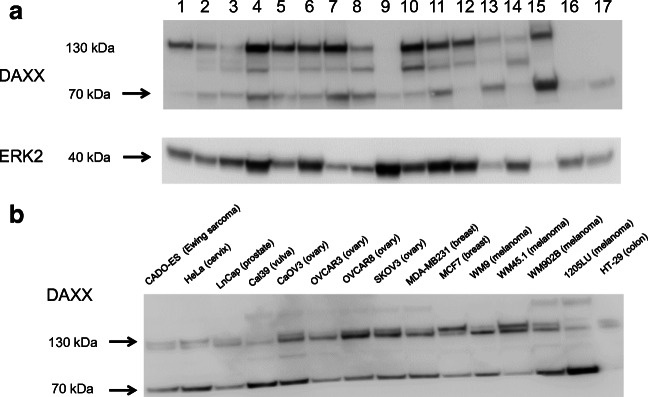
Western blotting: **a** DAXX expression in 17 effusion specimens; 14 specimens express DAXX at its full form (130 kDa), whereas all effusions have a band at 70 kDa (see text). ERK2 was used as housekeeping protein; **b** DAXX expression in cell lines. All 15 lines, including 4 of ovarian origin, express DAXX at its full 130 kDa form and as the 70 kDa fragment

In view of the results obtained in analysis focusing on clinical end-points (see below), WB analyzed exclusively DAXX expression. In agreement with the IHC data, DAXX was expressed in 70/81 (86%) HGSC effusions. In the majority of these specimens, DAXX bands were detected at 130 kDa, as predicted for the full protein, as well as smaller variants, mainly at 70 kDa. In few of the remaining specimens, only the 70 kDa band was detected (Fig. 2a). Both the 130 kDa and 70 kDa DAXX bands were additionally observed in all 15 cell lines analyzed, including carcinomas of ovarian (CaOV3, OVCAR3, OVCAR8, SKOV3), cervical (HeLa), vulvar (Cal39), breast (MDA-MB231, MCF7), colon (HT-29), and prostate (LnCap) origin, as well as 4 melanoma (WM9, WM45.1, WM902B, 1205 LU) and 1 Ewing sarcoma (CADO-ES) lines (Fig. 2b).

### DAXX expression in HGSC effusions is related to disease progression and survival

DAXX expression by IHC was higher in pleural compared to peritoneal effusions (*p* = 0.006) and in post-chemotherapy compared to pre-chemotherapy effusions (*p* = 0.004), with a marginally higher expression in FIGO stage IV compared to stage III disease (*p* = 0.05), whereas ATRX expression was unrelated to clinicopathologic parameters (Tables 2, 3).

**Table 2:** Association between DAXX expression and clinicopathologic parameters

Parameter		ATRX staining extent					*p* value
		0%	1–5%	6–25%	26–75%	76–100%	
Effusion site	Peritoneum (*n* = 343)	48	106	44	117	28	**0.006**
	Pleura (*n* = 57)	4	14	4	25	10	
Previous chemotherapy^a^	No (*n* = 263)	42	86	27	87	21	**0.004**
	Yes (*n* = 133)	10	33	21	54	15	
Age	≤ 60 (*n* = 164)	24	45	14	66	15	0.634
	> 60 (*n* = 236)	28	75	34	76	23	
FIGO stage^b^	III (*n* = 235)	38	69	30	80	18	0.05
	IV (*n* = 151)	14	47	15	55	20	
RD volume^c^	0 cm (*n* = 31)	5	11	4	11	0	0.547
	≤ 1 cm (*n* = 86)	13	26	10	24	13	
	> 1 cm (*n* = 93)	12	33	8	33	7	
Chemotherapy response^d^	Complete (*n* = 184)	33	55	17	63	16	0.08
	Other (*n* = 167)	14	53	21	61	18	

Bold marks statistically significant value

^a^For 396 patients, 4 patients with no data

^b^For 386 patients, 14 patients with stage I–II disease or no data

^c^For 210 patients who received upfront surgery

^d^For 351 patients, 49 patients with no data; other = partial response, stable disease or progressive disease

**Table 3:** Association between ATRX expression and clinicopathologic parameters

Parameter		ATRX staining extent					*p* value
		0%	1–5%	6–25%	26–75%	76–100%	
Effusion site	Peritoneum (*n* = 343)	11	33	33	152	114	0.873
	Pleura (*n* = 57)	3	6	6	20	22	
Previous chemotherapy^a^	No (*n* = 263)	11	28	30	108	86	0.172
	Yes (*n* = 133)	3	11	9	63	47	
Age	≤ 60 (*n* = 164)	6	13	15	73	57	0.522
	> 60 (*n* = 236)	8	26	24	99	79	
FIGO stage^b^	III (*n* = 235)	8	20	21	107	79	0.434
	IV (*n* = 151)	6	18	18	58	51	
RD volume^c^	0 cm (*n* = 31)	0	3	4	14	10	0.553
	≤ 1 cm (*n* = 86)	5	7	6	39	29	
	> 1 cm (*n* = 93)	3	12	14	36	28	
Chemotherapy response^d^	Complete (*n* = 184)	9	17	16	79	63	0.594
	Other (*n* = 167)	3	20	19	73	52	

^a^For 396 patients, 4 patients with no data

^b^For 386 patients, 14 patients with stage I–II disease or no data

^c^For 210 patients who received upfront surgery

^d^For 351 patients, 49 patients with no data; other = partial response, stable disease or progressive disease

The follow-up period ranged from 1 to 179 months (mean = 37 months, median = 29 months). PFS ranged from 0 to 148 months (mean = 11 months, median = 7 months). At the last follow-up, 357 patients were dead of disease, 27 were alive with disease, and 6 were with no evidence of disease. Five patients died of complications or other causes, 3 were lost to follow-up, and 2 had no survival data.

**Fig. 3:**
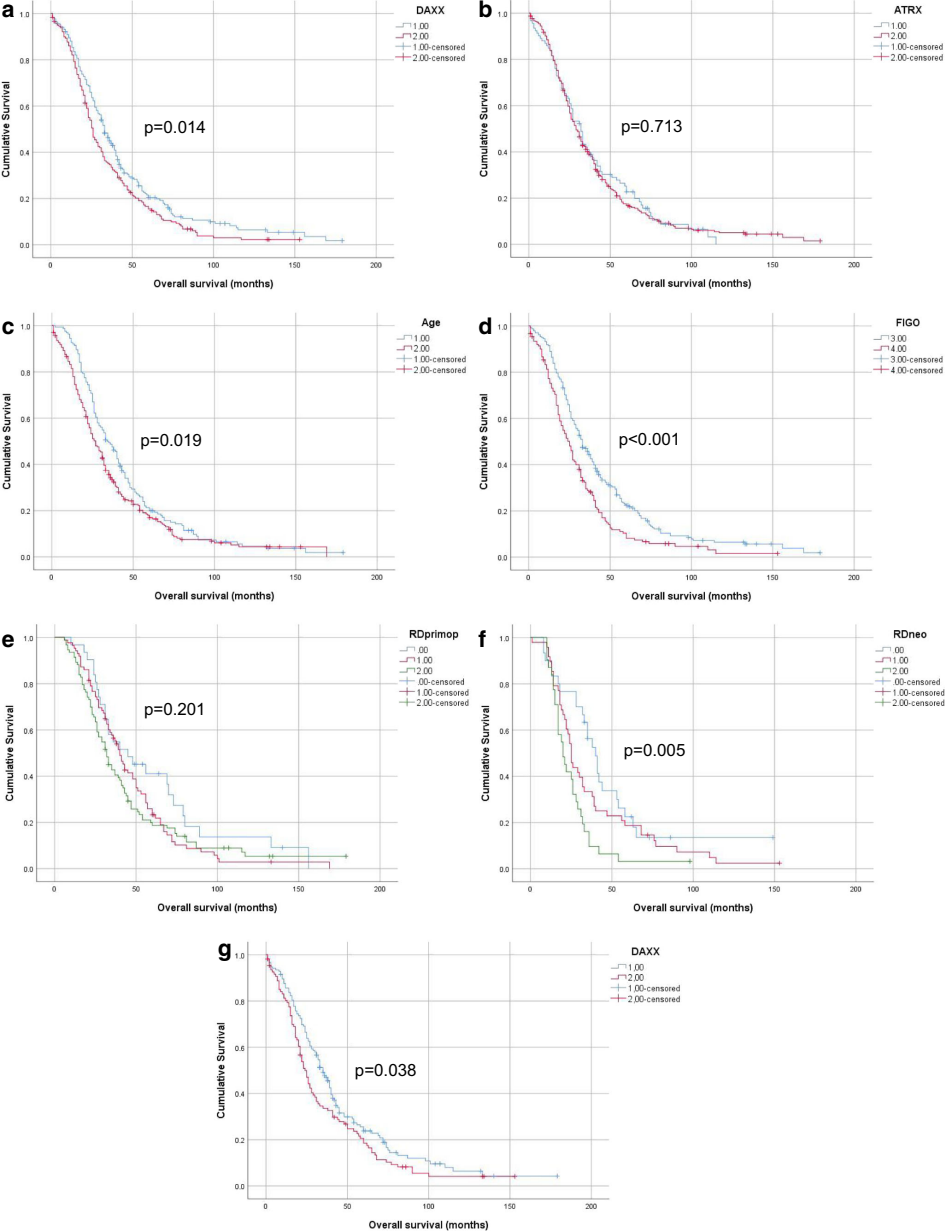
Survival: **a** Kaplan-Meier survival curve showing the association between DAXX protein expression and overall survival (OS) for 398 patients with survival data. Patients with effusions with high (> 25%) DAXX expression (*n* = 180; red line) had mean OS of 35.3 months compared to 44.8 months for patients with effusions having low (≤ 25%) DAXX expression (*n* = 218, blue line; *p* = 0.014). **b** Kaplan-Meier survival curve showing the association between ATRX protein expression and OS for 398 patients with survival data. Patients with effusions with high (> 25%) ATRX expression (*n* = 306; red line) had mean OS of 40.5 months compared to 39.6 months for patients with effusions having low (≤ 25%) ATRX expression (*n* = 92, blue line; *p* = 0.710.7133). **c** Kaplan-Meier survival curve showing the association between patient age and OS for 398 patients with survival data. Older (> 60 years) patients (*n* = 234; red line) had mean OS of 37.6 months compared to 45 months for younger (≤ 60 years) patients (*n* = 164, blue line; *p* = 0.019). **d** Kaplan-Meier survival curve showing the association between FIGO stage and OS for 386 patients with survival data and FIGO stage III–IV disease. Patients diagnosed with stage IV disease (*n* = 151; red line) had mean OS of 31.1 months compared to 45.2 months for patients with stage III disease (*n* = 235, blue line; *p* < 0.001). **e** Kaplan-Meier survival curve showing the association between residual disease (RD) volume and OS for 210 patients who received surgery as upfront treatment. Patients debulked to no macroscopic disease (*n* = 31; blue line) had mean OS of 59.7 months compared to 46.6 and 44.8 months for patients debulked to 1 cm (*n* = 86, red line) and ≥ 2 cm (*n* = 93, green line), respectively (*p* = 0.201). **f** Kaplan-Meier survival curve showing the association between RD volume and OS for 109 patients who received neoadjuvant chemotherapy prior to debulking surgery. Patients debulked to no macroscopic disease (*n* = 30; blue line) had mean OS of 50.7 months compared to 37.9 and 25.1 months for patients debulked to 1 cm (*n* = 48; red line) and ≥ 2 cm (*n* = 31; green line), respectively (*p* = 0.005). **g** Kaplan-Meier survival curve showing the association between DAXX protein expression and OS for 261 patients with pre-chemotherapy effusions and survival data. Patients with effusions with high (> 25%) DAXX expression (*n* = 108; red line) had mean OS of 36.2 months compared to 46.4 months for patients with effusions having low (≤ 25%) DAXX expression (*n* = 153, blue line; *p* = 0.038)

Higher DAXX expression was significantly associated with shorter OS in univariate analysis of the entire cohort (*p* = 0.014; Fig. 3a), with no such association for ATRX (*p* = 0.713; Fig. 3b). Among clinical parameters, older age (*p* = 0.019; Fig. 3c) and FIGO IV stage (*p* < 0.001; Fig. 3d) were significantly related to OS. RD volume was not significantly related to shorter OS in analysis of patients who received upfront surgery (*p* = 0.201; Fig. 3e), but was a prognosticator in analysis of patients who received neoadjuvant chemotherapy (*p* = 0.005; Fig. 3f). DAXX expression was additionally associated with shorter OS in analysis limited to chemo-naïve effusions tapped at diagnosis (*p* = 0.038).

The parameters entered in Cox multivariate survival analysis of the entire cohort were DAXX expression, age and FIGO stage. All 3 parameters retained their independent prognostic value in this analysis (DAXX, *p* = 0.011; age, *p* = 0.034; FIGO stage, *p* = 0.001).

DAXX expression was not significantly related to PFS (10.5 and 12.4 months for high and low expression; *p* = 0.095; data not shown).

## Discussion

Loss of ATRX or DAXX though mutation has been reported in cancers which are histogenetically remote from OC. Nevertheless, given the central role of these molecules in chromatin remodeling, and consequently in proliferation, analysis of the clinical relevance of ATRX and DAXX in HGSC was deemed to be of interest.

Our data suggest that ATRX and DAXX are diffusely expressed in the majority of HGSC in effusion specimens. Analysis of DAXX expression by WB further showed 2 principal forms of this protein, with band size of 130 kDa and 70 kDa. The presence of different splice variants of DAXX, termed DAXX-β and DAXX-γ, was previously reported by Wethkamp et al. in analysis of renal cell carcinoma cell lines by WB, followed by mRNA analysis of a panel of cell lines of other cancers [13]. However, this has not been previously reported in HGSC. The functional and clinical relevance of the 70 kDa isoform awaits further research. Of note, we previously reported on the association between the presence of cyclin E fragments and aggressive clinical behavior in OC [14].

In the present study, significantly higher DAXX expression was found in pleural compared to peritoneal effusions and in post-chemotherapy compared to pre-chemotherapy effusions, findings that suggest an association between this protein and disease progression in HGSC. DAXX was additionally significantly related to shorter OS, a finding that retained its independent prognostic relevance in Cox multivariate analysis.

DAXX and/or ATRX expression has been associated with both better and worse survival in analysis of other cancers [15–17]. No data regarding the clinical relevance of ATRX in OC is available to date, whereas data with respect to DAXX are to date limited to a single study by Pontikakis et al., in which analysis of 187 tumors divided into experimental and validation sets did not show any association between *DAXX* mRNA expression and survival. In addition to the obvious differences between analysis of mRNA levels and protein expression, and between primary tumors and metastatic disease, there are 2 crucial differences between the latter study and our study: Tumors in the Pontikakis series were of different histotypes and were not classified based on the WHO 2014 guidelines. In the present study, a uniform series of 400 HGSC, classified after the WHO 2014 criteria, was studied. OC are a heterogeneous group of tumors [18] and expression of biomarkers in the different histotypes has different clinical relevance, a fact that underscores the importance of studying each histotype separately.

In conclusion, the present study is the first to document a potential role for DAXX in mediating tumor progression and affecting outcome in metastatic HGSC, whereas ATRX expression does not appear to be informative in this tumor. The finding of splice variants of DAXX merits further research into potential differences in their biological and clinical relevance.
